# Rational design of novel sirtuin 1 activators via structure-activity insights from application of QSAR modeling

**DOI:** 10.17179/excli2019-1274

**Published:** 2019-04-05

**Authors:** Reny Pratiwi, Veda Prachayasittikul, Supaluk Prachayasittikul, Chanin Nantasenamat

**Affiliations:** 1 Center of Data Mining and Biomedical Informatics, Faculty of Medical Technology, Mahidol University, Bangkok 10700, Thailand; 2 Department of Medical Laboratory Technology, Faculty of Health Sciences, Setia Budi University, Surakarta 57127, Indonesia

**Keywords:** Sirtuin 1 activator, aging disease, Alzheimer's disease, QSAR, drug design, structural modification, cheminformatics

## Abstract

Sirtuin 1 (SIRT1) enzyme regulates major cell activities, and its activation offers lucrative therapeutic potentials for aging diseases including Alzheimer's disease (AD). Regarding the global aging society, continual attention has been given to various chemical scaffolds as a source for the discovery of novel SIRT1 activators since the discovery of the pioneer activator, resveratrol. Understanding structure-activity relationship (SAR) is essential for screening, designing as well as improving the properties of drugs. In this study, an *in silico *approach based on quantitative structure-activity relationship (QSAR) modeling, was employed for understanding the SAR of currently available SIRT1 fused-aromatic activators (i.e., imidazothiazole, oxazolopyridine, and azabenzimidazole analogs). Three QSAR models constructed using multiple linear regression (MLR) provided good predictive performance (*R^2^_LOOCV_* = 0.729 - 0.863 and RMSE*_LOOCV_* = 0.165 - 0.325). An additional novel set of 181 structurally modified compounds were rationally designed according to key descriptors deduced from the QSAR findings and their SIRT1 activities were predicted using the constructed models. In overview, the study provides insightful SAR findings of currently available SIRT1 activators that would be useful for guiding the rational design, screening, and development of further potent SIRT1 activators for managing age-related clinical conditions. A series of promising compounds as well as important scaffolds and molecular properties for potent SIRT1 activator were highlighted. This study demonstrated the efficacious role of QSAR-driven structural modification for the rational design of novel leads.

## Introduction

Silent information regulator 2 homolog one (Sirtuin 1 or SIRT1) is a member of class III histone deacetylases (HDACs) protein family that plays a major role in catalyzing the removal of acetyl group from acetyl-lysine substrates. Human SIRT1 is mainly found in the nucleus, but it is occasionally translocated into the cytoplasm (Haigis and Sinclair, 2010[18]; Jing and Lin, 2015[24]). Through the deacetylation of histone and other proteins, SIRT1 regulates a wide variety of important cellular processes including transcriptional silencing, cell cycle, and DNA damage responses (North and Verdin, 2004[37]). Due to the central role of SIRT1 in metabolic pathways and disease progression, considerable efforts have been directed towards the discovery of SIRT1 modulator as a novel approach for the development of disease-modifying therapy. Following the identification of resveratrol as a potent SIRT1 activator, a series of chemical entities (i.e., imidazothiazoles, oxazolopyridines, biphenyl and phenylhydrazones) have been developed as potent SIRT1-targeting candidates for the treatment of aging diseases (Bemis et al., 2009[3]; Blum et al., 2011[4]; Dai et al., 2010[8]; Wu et al., 2013[54]). 

Alzheimer's disease (AD) is an age-related disease that has become a major global health burden affecting more than 50 millions people in 2018 (WHO, 2018[50]). It is responsible for approximately 2 million of death and disability in 2016, making it the top ten leading causes of death worldwide (WHO, 2018[51]). Due to the rise of life expectancy and aging population, AD prevalence is predicted to be threefold increased by 2050 (WHO, 2018[50]). As the numbers and costs are increased in the coming years, and the fact that AD is currently untreatable, the development of innovative strategies to prevent, delay the progression, and cure AD has become a major research priority (Ballard et al., 2011[2]; Karagiannis and Ververis, 2012[25]). The hallmarks of AD include i) formation of amyloid β (Aβ) plaques resulted from an unusual cleavage of the amyloid precursor protein (APP) and ii) hyperphosphorylated tau protein tangles (Alzheimer's Association, 2018[1]). Normally, APP is cleaved by the α-secretase giving the neuroprotective soluble product, but the abnormal cleavage by β- and γ-secretases leads to the production of an insoluble Aβ peptides in which their accumulation leads to Aβ plaque formation (Ballard et al., 2011[2]). Due to the complexity of this multifactorial disease, the definitive understanding regarding the pathogenesis of AD is still unclear. Several hypotheses have been proposed. Among these, Aβ hypothesis considers the Aβ plaque as a causative agent leading to other pathological consequences, therefore, the inhibition of Aβ production is considered to be an attractive strategy with high therapeutic potential (Du et al., 2018[11]).

The association of SIRT1 with neuronal functions, and neurodegenerative diseases including AD has been recognized (Hou et al., 2016[21]; Kokkonen et al., 2014[28]). There is a relationship between methylation profiles that cause the silencing of SIRT1 gene and the severity of AD (Hou et al., 2013[20]). On the other hand, an increase of SIRT1 activity has been reported to reduce AD-like disorder in neuronal cell culture and in animal studies (Donmez, 2012[10]). Similarly, *in vitro* activation of SIRT1 by either NAD^+^ or the small molecule resveratrol has been shown to reduce the formation of Aβ oligomer by increasing the APP metabolism via the α-secretase (Braidy et al., 2012[5]). Moreover, the SIRT1 enhancing effect of the natural compound (resveratrol) gives a beneficial effect in extending the yeast lifespan (Howitz et al., 2003[22]). Taken together, these evidences have led to the growing research to explore the role of SIRT1 as a potential target for the development of novel therapeutics for AD (Braidy et al., 2012[5]). Currently, several classes of SIRT1 modulators have been experimentally identified (Bemis et al., 2009[3]; Kim et al., 2018[27]; Kumar et al., 2017[30]; Manna et al., 2018[32]; Vu et al., 2009[49]).

Computational tools have been employed to facilicate many stages of the drug discovery and development process to reduce time and cost as well as to increase the success rate (Prachayasittikul et al., 2015[42]). Quantitative structure-activity relationship (QSAR) is a computational method to establish correlation between the chemical structure and its bioactivity (Nantasenamat et al., 2009[35], 2010[36]), which is widely recognized as an effective method to rationally predict the bioactivity of compound and its mechanism of action (Shoombuatong et al., 2017[45]). QSAR models have been constructed to unveil the structure-activity relationship (SAR) of various classes of compounds and their biological activities (Diukendjieva et al., 2019[9]; Lomba et al., 2019[31]; Prachayasittikul et al., 2015[39], 2017[41]; Shoombuatong et al., 2015[44]; Simeon et al., 2016[46]; Worachartcheewan et al., 2012[53]). 

A search from the ChEMBL database revealed that there were 1,428 SIRT1 modulators and 2,474 bioactivity data points. Most compounds were reported as SIRT1 inhibitors, while less than a hundred were described as SIRT1 activators. This demonstrated that current situation in the development of SIRT1 activating compounds is still in its infancy mainly due to the lack of structural diversity.

Despite the limited number of small molecule identified as SIRT1 activators, some studies have exploited QSAR method to understand the SAR and their mechanisms (Chauhan and Kumar, 2018[6]; Karaman et al., 2016[26]; Kumar and Chauhan, 2017[29]; Park et al., 2009[38]). Each of the QSAR study has its own merit in encouraging this research area. However, an in-depth SAR analysis of the constructed *in silico* models to reveal the key chemical features is still essential for the discovery of novel potent SIRT1 activators, in terms of guiding their rational design and screening. Notably, these QSAR models were constructed from datasets comprising several classes of known SIRT1 activators without considering the diversity of their scaffolds. Scaffold refers to the structural core of a compound where functional groups (R groups) are attached (Hu et al., 2016[23]). The scaffold concept is widely applied in the field of medicinal chemistry. The concept concerns that each scaffold has its own characteristics and compounds with different core scaffolds are not truly be compared (Zdrazil and Guha, 2018[56]). In medicinal chemistry viewpoint, more effective SAR analysis could be achieved when compounds with the same core structures are compared (Hu et al., 2016[23]; Mok and Brown, 2017[34]). 

The aim of this study is to construct QSAR models using multiple linear regression (MLR) in order to achieve effective SAR insights of known SIRT1 activators. Compounds obtained from the ChEMBL database were separated into three sets according to their chemical core structures (i.e., imidazothiazole, oxazolopyridine, and azabenzimidazole) for QSAR modeling. Considering the limited numbers and the lack of structural diversity of available SIRT1 activators, an *in silico* structural modification of known SIRT1 activators was performed to expand its chemical space. Additional sets of structurally modified compounds were designed, and their activities were predicted using the constructed QSAR models followed by an in-depth SAR analysis. Finally, the built QSAR models were applied for predicting a set of novel SIRT1 activators to offer mechanistic interpretation of their mechanisms of action. 

## Materials and Methods

### Data set curation

A data set of known SIRT1 activators were collected from the ChEMBL database (Gaulton et al., 2012[14], 2017[15]) and curated according to the established protocol proposed by Fourches et al. (2010[12]). The main steps of data curation are as follows: (i) removal of inorganics and mixtures, (ii) structural conversion and cleaning, (iii) normalization of specific chemotypes, (iv) removal of duplicates, and (v) final manual checking.

An in-house script coded in the R statistical language was used to pre-process the initial data collected from the ChEMBL database. Briefly, data with missing SMILES notation, data containing < or > symbols, and duplicate data were removed. A final data set of 17 non-redundant compounds were attained along with their SMILES notation and bioactivity information (EC_1.5_). The experimental bioactivity was expressed as pEC_1.5_, which is the negative logarithmic form. EC_1.5 _values represented the concentration required to increase the SIRT1 enzymatic activity by 50 % (Bemis et al., 2009[3]; Milne et al., 2007[33]; Vu et al., 2009[49]). 

**Figure 1:**
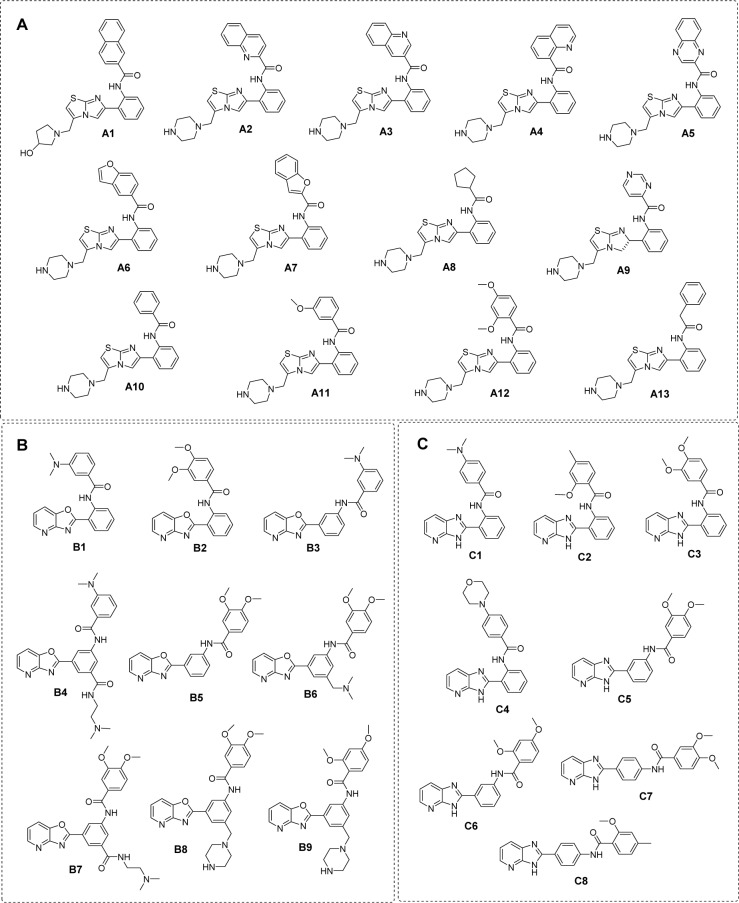
Chemical structure of SIRT1 activators (A) scaffold A: imidazothiazole, (B) scaffold B: oxazolopyridine, and (C) scaffold C: azabenzimidazole

**Figure 2:**
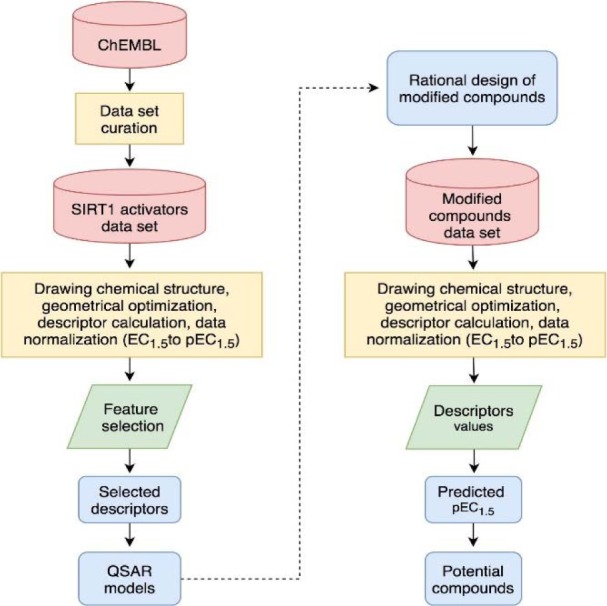
Workflow of the study

Finally, manual check of the reference source of the ChEMBL-derived data set was performed that resulted in the addition of 13 compounds that were not originally included in the aforementioned set of 17 compounds (Bemis et al., 2009[3]; Blum et al., 2011[4]; Milne et al., 2007[33]; Vu et al., 2009[49]; Wu et al., 2013[54]). Afterwards, the data set was divided into three groups with respect to their main scaffolds, namely scaffold A (imidazothiazole derivatives), scaffold B (oxazolopyridine derivatives), and scaffold C (azabenzimidazole derivatives). Final data sets consisted of compounds **A1**-**A13**, **B1**-**B9**, and **C1**-**C8 **belonging to scaffolds A, B, and C, respectively (Figure 1(Fig. 1)). A schematic workflow of the study is presented in Figure 2(Fig. 2).

### Molecular descriptors calculation

Chemical structures of the curated data set in the SMILES format were converted into the .mol format and further optimized using Gaussian 09 (Frisch et al., 2009[13]) to obtain low energy conformation. Geometrical optimization of all chemical structures were achieved by semi-empirical Austin Model 1 (AM1) level followed by density functional theory (DFT) computation using the Becke's three-parameter hybrid method with the Lee-Yang-Parr correlation functional (B3LYP) together with the 6-31 g(d) level. The optimized structures were used for calculation of the first set of thirteen quantum chemical descriptors using an in-house developed script: the mean absolute atomic charge (*Qm*), total energy (Etotal), total dipole moment (µ), highest occupied molecular orbital energy (HOMO), lowest unoccupied molecular orbital energy (LUMO), energy difference of HOMO and LUMO (HOMO-LUMOGap), electron affinity (EA), ionization potential (IP), Mulliken electronegativity (χ), hardness (η), softness (S), electrophilic index (ω i), and electrophilicity (ω). 

An additional set of 3,224 molecular descriptors were calculated using the Dragon software (version 5.5) (Talete, 2007[48]), including 22 blocks of following descriptors: Constitutional descriptors, Topological descriptors, Walk and path counts, Connectivity indices, Information indices, 2D autocorrelation, Edge adjacency indices, Burden eigenvalues, Topological charge indices, Eigenvalue-based indices, Randic molecular profiles, Geometrical descriptors, RDF descriptors, 3D-MoRSE descriptors, WHIM descriptors, GETAWAY descriptors, Functional group counts, Atom-centred fragments, Charge descriptors, Molecular properties, 2D binary fingerprints, and 2D frequency fingerprints.

### Descriptors selection

In order to select a set of informative descriptors from a large set of calculated descriptors, the correlation-based feature selection was employed. The Pearson's correlation coefficient (r) value of 0.5 was used as a cut-off for initial selection, following the calculation of pairwise correlation of each descriptor value and bioactivity (pEC_1.5_). Descriptors with |r| ≥ 0.5 were selected for additional selection process using stepwise multiple linear regression (MLR) in SPSS Statistics 18.0 software (SPSS Inc., USA[47]). The final set of informative descriptors and their values were selected for further QSAR model development. 

### QSAR model construction 

QSAR models were separately developed according to the three different scaffolds using MLR method implemented in Waikato Environment for Knowledge Analysis (WEKA) version 3.8 (Witten et al., 2011[52]) according to the equation (1).

*Y* = *B_0_* + Σ *B_n_X_n_* (1)

where *Y *is the pEC_1.5_ values of compounds, *B_0_* is the intercept and *Bn *are the regression coefficient of descriptors *X_n_*.

### Validation of QSAR models

Leave-one-out cross validation (LOO-CV) was employed to validate the predictive ability of constructed model. For small data sets of less than 50 compounds, LOO-CV represents a reliable method for QSAR model validation (Gramatica, 2007[16]; Hawkins, 2004[19]). The LOO-CV method was performed by removing one sample from the data set and used it as the testing set, while the remaining were used to build the QSAR model (Roy et al., 2015[43]). This cycle was repeated until every sample in the data set was used as the testing set. Furthermore, two statistical parameters were used to measure the predictive performance of the constructed QSAR models i.e., the squared correlation coefficient (*R^2^*) and root mean square error (RMSE) (Prachayasittikul et al., 2017[41]).

### Prediction of structurally modified compounds

To expand the chemical space of SIRT1 activators, a set of 181 structurally modified compounds (**A1a**-**A13c**, **B1a**-**B8a**, and **C1a**-**C8b**; Suppl. Figures 1-3) were rationally designed according to the QSAR results of known SIRT1 activators. These modified compounds were constructed *in silico* and their key descriptor values were obtained in a similar manner with those of the original compounds as mentioned above. Subsequently, the obtained descriptor values of modified compounds were used to predict the SIRT1 activity according to the QSAR equations. 

## Results and Discussion

### QSAR modeling of currently available SIRT1 activators

A set of informative descriptors was obtained using correlation-based feature selection. Definitions of the selected descriptors (Table 1(Tab. 1)) and descriptor values of the investigated compounds (Suppl. Tables 1-3) are presented. The equations (2-4) of constructed QSAR models and their predictive performance evaluation are shown in Table 2(Tab. 2). 

**Figure 3:**
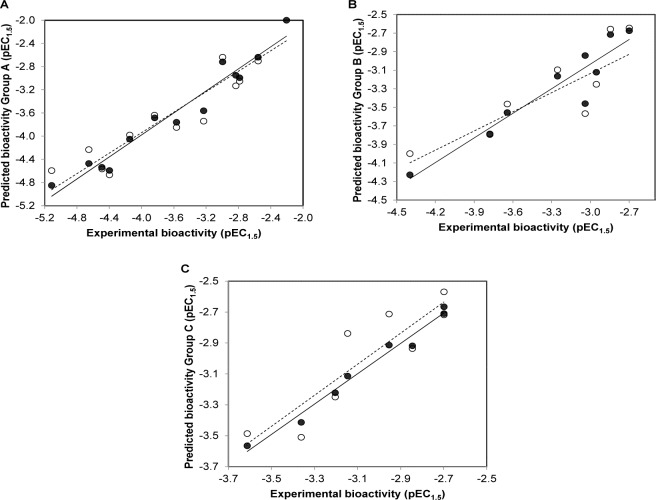
Plots of experimental versus predicted pEC_1.5_ values of SIRT1 activators (A) scaffold A, (B) scaffold B, (C) scaffold C generated by QSAR models (training set: compounds are denoted by black circle and regression line is shown as solid line; leave-one-out validated testing set: compounds are represented by open circle and regression line is shown as dashed line)

The QSAR models provided a good predictive performance for the training set as measured by *R^2^*_Tr_ values of 0.950, 0.884, and 0.980, and RMSE_Tr_ values of 0.197, 0.175, and 0.043, for scaffolds A, B, and C, respectively. Likewise, in the testing set, *R^2^_LOOCV_* values of 0.863, 0.729, and 0.800, and RMSE*_LOOCV_* values of 0.325, 0.271, and 0.165, were noted for scaffolds A, B, and C, respectively. The experimental and predicted bioactivities of SIRT1 activators (**A1**-**A13**, **B1**-**B9**, and **C1**-**C8**) are summarized in Table 3(Tab. 3) and Figure 3(Fig. 3). 

The QSAR model of scaffold A (Table 2, Eq. 2(Tab. 2)) revealed that electronegativity, charge, and polarizability influenced the bioactivity of imidazothiazole analogs. The charge index descriptor (JGI7) was the most influential descriptor as shown by its highest regression coefficient value of 593.47. Accordingly, the high value of JIGI7 along with the low values of HATS8u, Electronegativity, and polarizability descriptor Mor15p are required for potent activity of the compounds. The most potent compound **A5** displayed the lowest HATS8u value together with high Electronegativity (HATS8u = 0.165, Electronegativity = -0.136, Suppl. Table 1). 

The QSAR analysis of scaffold B (Table 2, Eq. 3(Tab. 2)) showed that activities of the oxazolopyridine derivatives are mainly governed by electronegativity, as shown by the high regression coefficient values of both P1e (-2.1032) and Mor22e (-0.9163). It is noted that lower electronegativity values but higher frequency of C-O are desired for greater bioactivity. This can be seen when comparing the most potent compound **B9 **(pEC_1.5 _= -2.699 (Table 3(Tab. 3)), P1e = 0.665, Mor22e = -0.057, and F10[C-O] = 11, Suppl. Table 2) with the least active compound **B3 **(pEC_1.5 _= -4.398 (Table 3(Tab. 3)), P1e = 0.874, Mor22e = 0.325, F10[C-O]= 3, Suppl. Table 2) of the series.

The QSAR result of scaffold C (Table 2, Eq. 4(Tab. 2)) revealed that the descriptors related to mass (RDF090m and R6m+), atomic polarizabilities (BEHp2), and WHIM index (E2u) influenced the bioactivity of azabenzimidazole compounds. It is observed that the lower BEHp2 and E2u, but the higher values of RDF090m and R6m+ are important for potent SIRT1 activity. The most potent compound **C3** (pEC_1.5 _= -2.699, Table 3(Tab. 3)) displayed lower BEHp2 (3.816) and E2u (0.406), but higher RDF090m (8.575) and R6m+ (0.016) when compared with the least active compound **C2 **(higher BEHp2 = 3.836 and E2u = 0.452, but lower RDF090m = 4.688 and R6m+ = 0.014). Similarly, the most potent compound **C6 **(with an equivalent pEC_1. 5_ value) showed the same trend of lower and higher descriptor values (Suppl. Table 3).

### Application of QSAR models for rational design and in silico prediction of novel SIRT1 activators

The constructed models were further applied for the efficacious rational design of a novel set of 181 structurally modified compounds with relevant scaffolds. Key descriptors presented in the models revealed important features for guiding the structural modification strategy. Finally, three additional sets of structurally modified compounds were designed (109, 51, and 21 modified compounds for scaffolds A, B and C, respectively, Suppl. Figures 1-3), and their key descriptor values were calculated and subsequently applied to the QSAR equations for predicting their activities (Suppl. Tables 4-6). As a result, a set of promising novel compounds with the most potent predicted activities were highlighted for their potential for further development as SIRT1 activators i.e., compounds **A5d**, **B7a**, and **C4d **as shown in Suppl. Figures 1-3, (predicted pEC_1.5 _= -0.697, -1.589, and -1.948, resepectively, Suppl. Tables 4-6). 

### Understanding structure-activity relationships (SAR)

**Figure 4:**
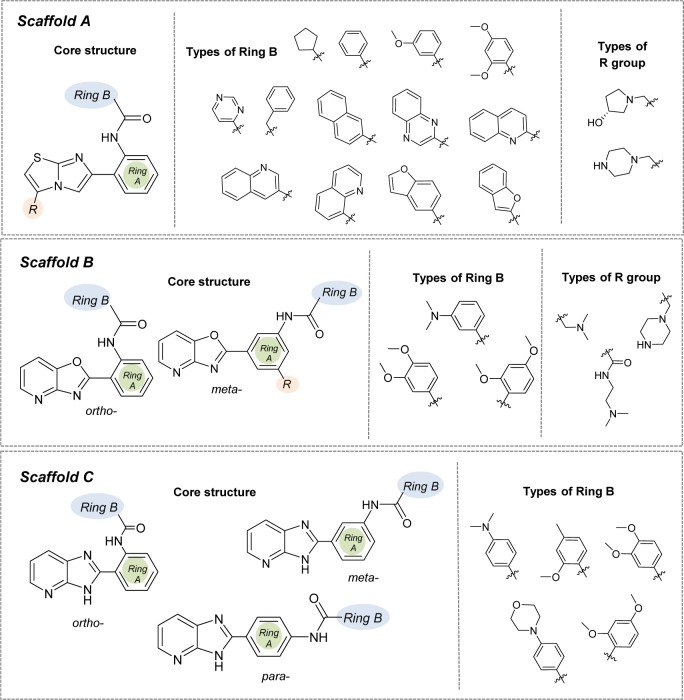
Structural modifications of compounds in the scaffolds A, B, and C (substitution with -OH, -OCH_3_, -NH_2_, and -SH groups at different positions of Ring B)

According to the QSAR results, an in-depth SAR analysis was performed considering the chemical descriptors governing bioactivity of the original (scaffolds A, B, and C, Suppl. Tables 1-3) and modified SIRT1 activators (Suppl. Tables 4-6). Compounds from the scaffolds A, B, C have a common amide group (Figure 4(Fig. 4)) as a linker of rings A and B, mostly, both are aromatic/substituted aromatics. 

#### Scaffold A

Scaffold A consists of 13 original compounds (**A1-A13) **with identical structural feature of fused imidazothiazole substituted on the ring A, which is connected to the ring B by an amide linker. The imidazothiazole ring of compounds **A2-A13 **was substituted with R group (piperazine), while compound **A1 **was substituted with R = pyrrolidinol (Figure 4(Fig. 4)). All of these compounds (**A1-A13**, Figure 1(Fig. 1)) had three different types of ring B including aromatic and hetero-aromatic fused rings (**A1-A7**), phenyl, benzyl, and pyrimidine rings (**A9-A13**), as well as cycloalkane ring (**A8**). The enzyme activation assay results showed that compound **A5 **(Milne et al., 2007[33]) exerted the most potent activity (pEC_1.5 _= -2.204) when compared with **A8** (Wu et al., 2013[54]) that showed the lowest activity (pEC_1.5 _= -5.114). 

Compounds from scaffold A (except for **A8)**, were structurally modified at various positions on the ring B by substitution of groups with lone pair electrons (OH, OMe, NH_2_, SH) to provide 109 modified compounds (Suppl. Figure 1). The results showed that ring B (naphthalene) of **A1** substituted by OH, OMe, NH_2_, and SH groups at the 6-position gave modified compounds with ranked activities as **A1b > A1d > A1a > A1c** which is relative to their substituted groups (OMe > SH > OH > NH_2_). When the naphthalene ring was substituted at the 8-position, the predicted activity was shown to be **A1h > A1e > A1f > A1g** (SH > OH > OMe > NH_2_). As a result, a series of compounds with higher potency were noted for **A1a-A1d** in which substitution at the 6-position gave rise to better activity than that afforded by the 8-position of naphthalene ring B. Compound **A1b** showed the most improved activity (pEC_1.5 _= -1.703) having high JGI7 (0.013) but low HATS8u (0.182) when compared with the parent compound **A1 **(pEC_1.5 _= -2.556, JGI7 = 0.011, HATS8u = 0.19). 

Compound **A2** was similarly modified by substituting ring B with OH, OMe, NH_2_, and SH groups to give compounds **A2a**-**A2h**. It was found that 2-quinolinyl (ring B) substituted at the 6-position resulted in compounds with better activity than that of the 8-position. All compounds exhibited increased activity with the same order of substituted group (SH > OH > OMe> NH_2_). The most potent compound **A2d** showed improvement of the predicted activity (pEC_1.5_ =-1.548, high JGI7 = 0.012, low HATS8u = 0.171, and electronegativity = -0.12861), when compared with the parent compound **A2** (pEC_1.5_ = -2.996, low JGI7 = 0.010, high HATS8u = 0.176, and electronegativity = -0.12731). 

In the case of compound **A3, **its 3-quinolinyl ring B was substituted at the 7-position to afford compounds with more improved activity (i.e. **A3d**) than that of the 5-position (**A3h**). The modified compound **A3d** was the most potent one (pEC_1.5 _= -1.387, low HATS8u = 0.178) when compared with the parent compound **A3 (**pEC_1.5 _= -2.833, high HATS8u = 0.260). The effect of substituted groups was ranked as SH > OMe > OH > NH_2_. In a series of **A4 **(pEC_1.5 _= -4.653) modified compounds, 8-quinolinyl (ring B) was substituted at 2-, 4-, 5-, and 7- positions. The results showed that the SH group substitution gave the most improved activity in all cases. Interestingly, substitution at the 5- position resulted in the most improved activity (**A4l**, predicted pEC_1.5 _= -1.107) with lower values of Mor15p (0.824) and electronegativity (-0.13489), but higher JGI7 (0.014) value compared with **A4** (pEC_1.5 _= -4.653, with Mor15p = 1.126, electronegativity = -0.12759, and JGI7=0.009).

**Figure 5:**
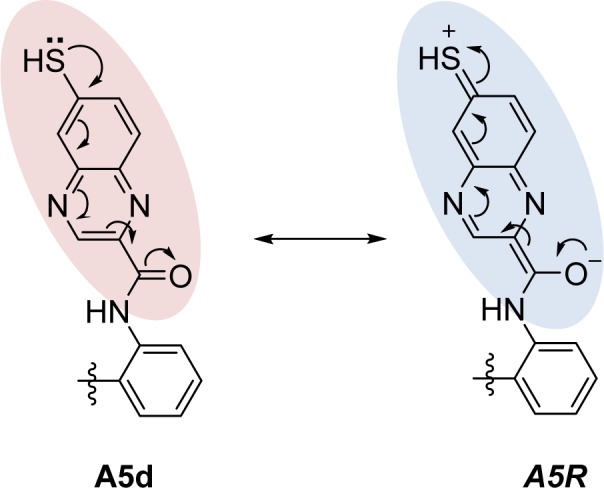
Resonant ionic formed (*A5R*) of the modified compound A5d

Ring B as benzopyrazine (**A5**, pEC_1.5 _= -2.204) was modified at the 6- and 8- positions resulting in compounds **A5a-A5h**. The result revealed that substitution at the 6- position (**A5d**) gave better activity than that of the 8-position. The SH substituted group displayed the most improved activity in a series of **A5a-A5d **(SH > OH >OMe > NH_2_) as compared with a series of **A5e-A5h **(OH > NH_2 _> OMe > SH), in which the SH group exerted the lowest predicted activity. In the **A5 **series, compound **A5d **(predicted pEC_1.5 _= -0.697) was shown to be the most potent one when compared with the other parent compounds (**A1-A4 **and **A6-A13**). Apparently, the most potent parent compound **A5 **gave rise to the most potent modified compound **A5d**. In particular, structural modification of **A5** at the 6-position provided a series of compounds, which ranked as the top 1, 2, 3, and 5 of all modified compounds as **A5d**,** A5a**,** A5b**, and** A5e**, respectively. The **A5d** displayed lower values of HATS8u (0.15) and electronegativity (-0.137) but higher JGI7 (0.012) when compared with the parent compound **A5 **(HATS8u = 0.165, electronegativity = -0.13634, and JGI7 = 0.010). This could be due to the presence of electron donor SH group at the 6-position of ring B (**A5d**) that provided a resonant ionic formed (***A5R***) resulting from an inductive effect of the carbonyl amide linker (Figure 5(Fig. 5)). Thus, the compound **A5d** with high mean topological charge index of order 7 (JGI7) was noted. 

Compound **A6 **was modified by substitution at positions 4- and 6- of 5-benzofuranyl ring B. The results displayed that **A6a** (substitution at 4- position) was the most potent compound from the series (**A6a > A6c > A6b > A6d**, with OH > NH_2 _> OMe > SH, respectively). In the case of 2-benzofuranyl ring B (compound **A7**, pEC_1.5 _= -2.785), substitution at 4- and 6- positions were performed to give **A7h **(predicted pEC_1.5 _= -2.282) as the most potent compound of the 6-substituted ring B (**A7h > A7g > A7f > A7e**, with SH > NH_2 _> OMe >OH, respectively). Compound **A9 (**pEC_1.5 _= -3.230) with a single ring B (pyrimidine) was replaced by a pyrazine ring (**A9a**) which was substituted at 3- and 5- positions. It was found that the 5- substitution gave the most potent **A9i **(pEC_1.5 _= -1.932) of the series **A9i **> **A9h > A9f > A9g **(SH > NH_2 _> OH > OMe). However, the pyrazine ring B (**A9a**) was less active than that of the pyrimidine ring B (**A9**).

Compound **A10 **(pEC_1.5 _= -4.398), ring B (phenyl) was substituted at 2-, 3- and 4- positions. Modified compounds **A10a-A10l** were obtained, in which substitution by OH, OMe, SH, NH_2_, at 3- and 4- positions yielded more improved activity as compared with **A10**. When the phenyl (ring B) was changed to 2- and 3-furanyl rings (B), **A10q** (SH at the 5- position of 2-furanyl ring B) provided the most potent predicted activity (pEC_1.5 _= -3.054) from the modified **A10 **series. Compound **A11 **with pEC_1.5 _= -3.568 (3-OMe derivative of **A10**) was modified by OH, OMe, NH_2_, SH substitutions at the position-6 of ring B (at *p*-position to the 3-OMe group) provided **A11a-A11d**, in which **A11a** (pEC_1.5 _= -3.353) was the most potent compound. Compound **A12 **(2,4-dimethoxyphenyl ring B), its 4-OMe group was replaced by OH, NH_2_, and SH groups. The results showed that the SH group (**A12c**) exerted the most potent activity (predicted pEC_1.5 _= -3.473). Benzyl ring B of compound **A13 **was modified by replacing phenyl with 2-, 3-, and 4-pyridyl rings to afford compounds **A13a-A13c**. The 4-pyridyl derivative **A13c **was the most potent one (predicted pEC_1.5 _= -3.361). 

All of the modified compounds from scaffold A (i.e., **A1a**-**A13c**, Suppl. Figure 1) displayed the improved activity when compared with their parent compounds. 

#### Scaffold B

Scaffold B is a series of compounds **B1-B9 **bearing ring A and ring B linked by the amide bond (Figure 4(Fig. 4)), where the ring A is either *ortho-* or *meta-*isomer, and ring B is phenyl substituted by amino and methoxy groups. Aryl group on ring A is a fused pyridooxazole ring (**B1-B3**, **B5**). It was noted that the *ortho- *series exerted more potent bioactivity than the *meta-*isomer (**B1 **> **B3** and **B2 **> **B5**). An extra side chain on the ring A gave rise to the compounds **B4**, **B6**,** B7**,** B8**, and** B9** as 1,3,5-trisubstituted ring A, in which **B9** (pEC_1.5 _= -2.699) was the most potent compound. On the other hand, 1,3-disubstituted ring A (**B3**) displayed the lowest activity (pEC_1.5 _= -4.398). It should be noted that ring B with 2,4-dimethoxy groups was more potent than 3,4-dimethoxy as noted for compounds **B9 **> **B8**. 

Furthermore, to achieve the improved activity, compounds (**B1-B9**) were structurally modified (Suppl. Figure 2). The 3-NMe_2_ group on ring B of compound **B1** was replaced by OH, OMe, NH_2_, and SH at 2-, 3-, and 4-positions to provide compounds **B1c-B1n**. Notably, **B1d** with 2-methoxy substitution on ring B was predicted as the most improved activity (pEC_1.5 _= -3.101) among the modified compounds in series **B1**. On the other hand, **B1f** (2-SH ring B, pEC_1.5 _= -3.761) displayed the lowest activity in the **B1** series. Compound **B3** was similarly modified as **B1** to obtain **B3a-B3n**, in which **B3c** (pEC_1.5 _= -3.585, P1e = 0.822, Mor22e = 0.055, F10[C-O] = 6) was the most active compound, but **B3k** (pEC_1.5 _= -4.134, P1e = 0.849, Mor22e = 0.279, F10[C-O] = 3) exhibited the lowest activity. 

The results showed that the *ortho*-ring A and 2-methoxy ring B (**B1d**) exerted higher activity than the *meta-*ring A and 2-methoxy ring B (**B3c**). Similarly, compounds **B2** and **B5** with 3,4-dimethoxy ring B were modified at ring B as 2,4-dimethoxy (**B2a** and **B5a**, pEC_1.5 _= -2.845 and -2.899) and 3,5-dimethoxy (**B2b** and **B5b**, pEC_1.5 _= -3.242 and -3.692), respectively. It was shown that the modified 2,4-dimethoxy exerted higher activity than the 3,5-dimethoxy compounds both in the *ortho*- and *meta-*isomers. Compound **B4**, its 3-NMe_2_ at ring B was changed to 2-NMe_2_ ring B (**B4a** > **B4**), which was replaced by OH, OMe, NH_2_, and SH groups at positions 2-, 3-, and 4- on the ring B to give compounds **B4b-B4h**. The most potent compound in this series was **B4h** with 4-methoxy ring B (pEC_1.5 _= -2.251). 

**Figure 6:**
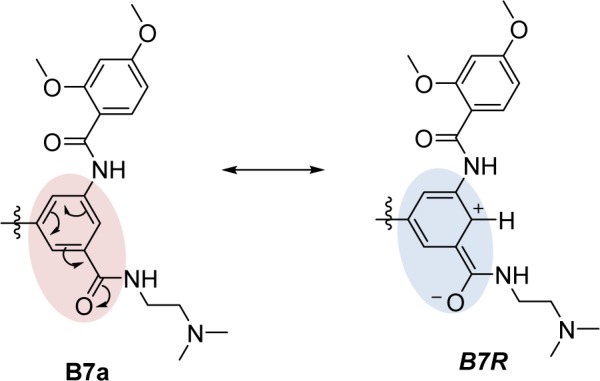
Resonant ionic formed (*B7R*) of modified compound B7a

Compounds **B6-B8** as 1,3,5-trisubstituted ring A and 3,4-dimethoxy ring B, which were modified at ring B as 2,4- and 3,5-dimethoxy to give compounds **B6a**, **B6b**, **B7a**, **B7b**, **B8a**. Compound **B7a** (2,4-dimethoxy ring B) was the most potent compound (pEC_1.5 _= -1.589, P1e = 0.603, Moer22e = -0.786, F10[C-O] = 14), and more potent than the parent compound **B9**. Notably, the **B9** and **B7a** had a similar substitution pattern on the ring B (2,4-dimethoxy) and ring A (1,3,5-trisubstituted). The ring A of both compounds has two identical substituents, but the third substituent of **B9** as piperazine ring and of **B7a** as amide side chain. The results revealed that **B7a** had lower electronegativity (P1e = 0.603, Mor22e = -0.786), but with higher frequency of [C-O] at topological distance of 10 (F10[C-O] = 14) compared with that of **B9** (P1e = 0.665, Mor22e = -0.057, F10[C-O] = 10). The higher frequency F10[C-O] of **B7a** might be resulted from the inductive effect of the carbonyl amide (CON) side chain substituted on the ring A as shown by its resonant ionic formed (***B7R***, Figure 6(Fig. 6)).

#### Scaffold C

Scaffold C compounds (**C1-C8**) were sub-classified into 3 subtypes according to the position of amide linker on the phenyl ring A of the core structure (i.e. *ortho*-, *meta-*, and *para-*series). Considering the *ortho* series (**C1-C4**), the most potent activity was obtained when the ring B was substituted by 3,4-dimethoxy groups (**C3**; pEC_1.5_ = -2.699), whereas the lower activity was observed for 4-dimethylamino (**C1**) and 4-morpholine (**C4**) substitutions on the ring B. 

The *meta-*series (**C5 **and **C6**) showed that compound **C6 **with 2,4-dimethoxy ring B exhibited the most potent activity (pEC_1.5 _= -2.699) as observed for 3,4-dimethoxy ring B (*ortho-*isomer **C3**). It was found that ring B with 4-methyl, instead of 4-methoxy, provided the compound with lower activity as noted for compounds **C2 **and **C8** (2-methoxy, 4-methyl) compared with 2,4-dimethoxy compound **C6** (the most potent). Both compounds (**C2 **and **C8, **pEC_1.5_ = -3.613 and -3.362, respectively) displayed the lowest activity (**C8 > C2**) amongst compounds from the scaffold C. Results indicated that the oxy function at position 4 of ring B may affect the activity of the compound through mass descriptor (RDF090m) value. Obviously, 3,4-dimethoxy ring B of *ortho-*isomer (**C3**) exerted the most potent activity than that of *meta*-isomer (**C5**) and *para-*isomer (**C7**) in which their ranked activities were observed as **C3 > C5 > C7**. 

The activity of compounds in this series was ranked as **C3 = C6 > C1 > C4 > C5 > C7 > C8 > C2**. The compounds in scaffold C (**C1**,** C3**,** C4**,** C6-C8**) were structurally modified by changing substituents (using OH, OMe, NH_2_, and SH groups), and other types of rings on the ring B as shown in Suppl. Figure 3. It was found that compound **C4d** was the most potent one (pEC_1.5 _= -1.948) and **C3a **with the lowest activity (pEC_1.5 _= -3.085). While, other modified compounds of **C4 **(**C4a**,** C4c**,** C4f**,** C4b**) were ranked as 2^nd^, 3^rd^, 5^th^, and 6^th^ from the highest predicted bioactivity. 4-Dimethylamino group on ring B of **C1 **was replaced by OH, OMe, NH_2_, and SH groups. All modified compounds exerted improved activity when compared with the parent compound **C1**. The most potent compound **C3 **(pEC_1.5 _= -2.699), its 3,4-dimethoxy ring B was modified as monomethoxy, i.e. 2-methoxy (**C3a**) and 3-methoxy (**C3b**), and dimethoxy, i.e. 2,4-dimethoxy (**C3c**) and 3,5-dimethoxy (**C3d**). Results showed that **C3b** was the only compound that possessed an improved activity (pEC_1.5 _= -2.305) when compared with **C3** (the most potent compound in the scaffold C). It is suggested that the high value of RDF090m (17.39) plays an essential role in improving the activity of compound **C3b**. On the other hand, 2-methoxy compound (**C3a**, with low RDF090m = 8.376) displayed the lowest activity (pEC_1.5 _= -3.085). 

Ring B of **C4** was modified by changing the 4-morpholine group to other ring types (such as piperazine and piperidine rings), and/or substituted with OMe group at various positions. All modified compounds (**C4a-C4h**) displayed improved activity (pEC_1.5 _= -2.812 to -1.948) when compared with the parent compound **C4 **(pEC_1.5 _= -2.954), and **C4d** was the most potent compound with the highest value of RDF090m (28.043) amongst the modified compounds from scaffold C. The improved effects were also observed for modified compounds following this order; **C7b > C7a > C7**, and** C8b > C8a > C8**. On the other hand, the most potent 2,4-dimethoxy ring B (**C6**) was modified to the 3,5-dimethoxy ring B (**C6a**), which led to lower activity (pEC_1.5 _= -2.848, high E2u = 0.448) when compared with the parent compound **C6 **(pEC_1.5 _= -2.699, low E2u = 0.381). 3,4-Dimethoxy ring B of **C7 **(ring A, *para-*isomer) was modified to 2-methoxy (**C7a**) and 3-methoxy (**C7b**) ring B leading to the improved activity of compounds (**C7b > C7a > C7**). In addition, the similar improved effect was noted for **C8 **(**C8b > C8a > C8**). High RDF090m value of the most potent modified compound **C4d** may be a result of the combination effects of OMe and piperazine ring substituted on the ring B.

**Figure 7:**
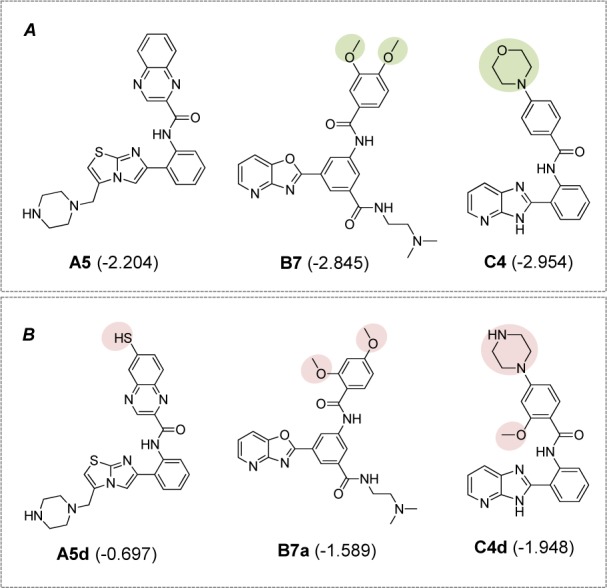
Potential novel compounds for further development as SIRT1 activators (A) Known SIRT1 activators (experimental pEC_1.5_), (B) Novel QSAR-driven SIRT1 activators (predicted pEC_1.5_)

Notably, all modified compounds in scaffolds **A**,** B**, and **C **displayed improved bioactivity when compared with their parent compounds. The most potent modified compounds (**A5d**,** B7a**, and** C4d**, Figure 7(Fig. 7)) are highlighted as potential novel SIRT1 activators to be further developed. 

## Conclusion

Understanding the SAR is considered to be a fundamental part of success drug discovery (Guha, 2013[17]). In this study, QSAR modeling and in-depth analysis were performed to gain insights into the SAR of available SIRT1 activators. Three QSAR models were successfully constructed with good predictive performance affording *R^2^_LOOCV_* ranging from 0.729 - 0.863 and RMSE*_LOOCV_* ranging from 0.165 - 0.325. The QSAR models revealed a set of important descriptors influencing the bioactivity of SIRT1 activating compounds including electronegativity, charge, polarizability, frequency of [C-O], and mass descriptors. 

According to the limited diversity of the currently available SIRT1 activators, an *in silico* structural modification was performed based on the key descriptors obtained from the QSAR analysis. Structural modification has been extensively used to obtain potential lead compounds with improved potency and pharmacokinetic properties, reduced toxicities (Chen et al., 2015[7]; Yao et al., 2017[55]), as well as considered to be an effective strategy for increasing the structural diversity (Prachayasittikul et al., 2014[40], 2015[39], 2017[41]). Herein, the constructed QSAR models were used to examine the effects of structural modifications on the bioactivity of SIRT1 activating compounds. A set of structurally modified compounds were virtually designed based on the key descriptors identified from the QSAR analysis, and their SIRT1 activities were predicted using the constructed QSAR models. In summary, the study provides insightful SAR findings that are beneficial for guiding the screening, rational design, and optimization of the relevant SIRT1 activating compounds. Of note, the study demonstrated successful application of the QSAR-driven rational design for discovery of new leads. Finally, a set of promising compounds were highlighted as potential SIRT1 activators (Figure 7(Fig. 7)) to be further developed for Alzheimer's disease, other aging diseases, and other relevant therapeutics. 

## Supplementary information

Supplementary information is available on the EXCLI Journal website.

## Acknowledgements

We gratefully acknowledge the support from the annual budget grant (B.E. 2562-2563) of Mahidol University and the scholarship to RP from the Directorate General of Resources for Science Technology and Higher Education of the Republic of Indonesia.

## Conflict of interest

The authors declare that they have no conflicts of interest.

## Supplementary Material

Supplementary information

## Figures and Tables

**Table 1:**
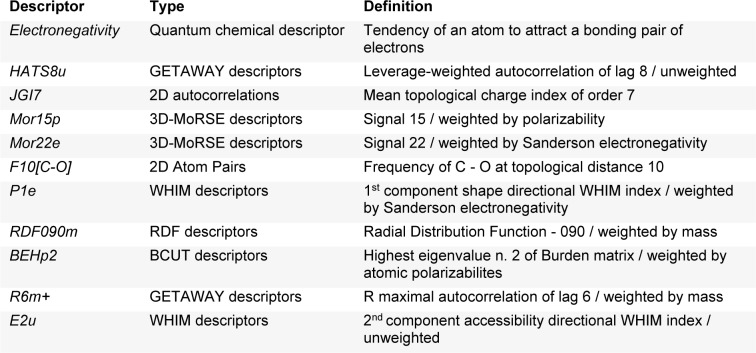
Definition of descriptors for construction of QSAR models

**Table 2:**
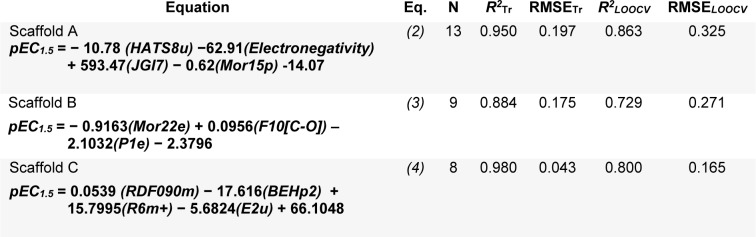
Summary of QSAR models and their predictive performance

**Table 3:**
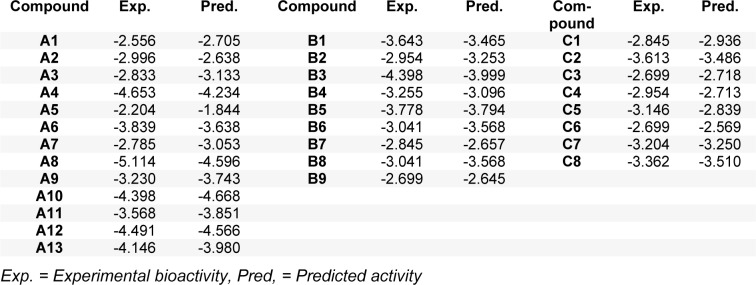
Experimental and predicted bioactivities (pEC_1.5_) of scaffolds A, B, and C
