# Supplementary material for: Rational design of novel sirtuin 1 activators via structure-activity insights from application of QSAR modeling
Source: EXCLI J. 2019 Apr 5;18:207–22. doi: 10.17179/excli2019-1274 (PMC6558509; doi:10.17179/excli2019-1274)
Supplement: Supplementary information [file EXCLI-18-207-s-001.pdf]

EXCLI Journal 2019;18:207-222 – ISSN 1611-2156
Received: March 12, 2019, accepted: March 20, 2019, published: April 05, 2019

Supplementary information:

RATIONAL DESIGN OF NOVEL SIRTUIN 1 ACTIVATORS VIA STRUCTURE-ACTIVITY INSIGHTS FROM APPLICATION OF QSAR MODELING

Reny Pratiwi[1,2], Veda Prachayasittikul[1,*], Supaluk Prachayasittikul[1], Chanin Nantasenamat[1]

[1] Center of Data Mining and Biomedical Informatics, Faculty of Medical Technology, Mahidol University, Bangkok 10700, Thailand
[2] Department of Medical Laboratory Technology, Faculty of Health Sciences, Setia Budi University, Surakarta 57127, Indonesia

* Corresponding author: Phone: +66 2 441 4371; Fax: +66 2 441 4380
E-mail: veda.pra@mahidol.ac.th

http://dx.doi.org/10.17179/excli2019-1274

This is an Open Access article distributed under the terms of the Creative Commons Attribution License (http://creativecommons.org/licenses/by/4.0/).

Supplementary Table 1: Descriptors values of SIRT1 activators Scaffold A

Compound	HATS8u	Electronegativity	JGI7	Mor15p
**A1**	0.190	-0.122	0.011	1.148
**A2**	0.176	-0.127	0.010	1.107
**A3**	0.260	-0.139	0.010	1.170
**A4**	0.284	-0.128	0.009	1.126
**A5**	0.165	-0.136	0.010	1.052
**A6**	0.197	-0.114	0.010	0.973
**A7**	0.186	-0.122	0.010	0.786
**A8**	0.289	-0.114	0.009	0.289
**A9**	0.243	-0.130	0.009	0.652
**A10**	0.252	-0.116	0.009	0.710
**A11**	0.196	-0.114	0.010	1.058
**A12**	0.266	-0.112	0.010	0.970
**A13**	0.259	-0.115	0.010	0.520

EXCLI Journal 2019;18:207-222 – ISSN 1611-2156
Received: March 12, 2019, accepted: March 20, 2019, published: April 05, 2019

Supplementary Table 2: Descriptors values of SIRT1 activators Scaffold B

Compound	Mor22e	F10[C-O]	P1e
**B1**	0.197	2	0.565
**B2**	-0.191	4	0.618
**B3**	0.325	3	0.874
**B4**	0.269	6	0.529
**B5**	0.033	5	0.882
**B6**	-0.320	6	0.680
**B7**	-0.009	9	0.573
**B8**	0.224	6	0.689
**B9**	-0.057	11	0.665

Supplementary Table 3: Descriptors values of SIRT1 activators Scaffold C

Compound	RDF090m	BEHp2	R6m+	E2u
**C1**	8.004	3.822	0.014	0.413
**C2**	4.688	3.836	0.014	0.452
**C3**	8.575	3.816	0.016	0.406
**C4**	9.007	3.830	0.014	0.397
**C5**	6.355	3.818	0.011	0.436
**C6**	7.724	3.819	0.016	0.381
**C7**	6.447	3.819	0.014	0.461
**C8**	4.961	3.839	0.013	0.416

EXCLI Journal 2019;18:207-222 – ISSN 1611-2156
Received: March 12, 2019, accepted: March 20, 2019, published: April 05, 2019

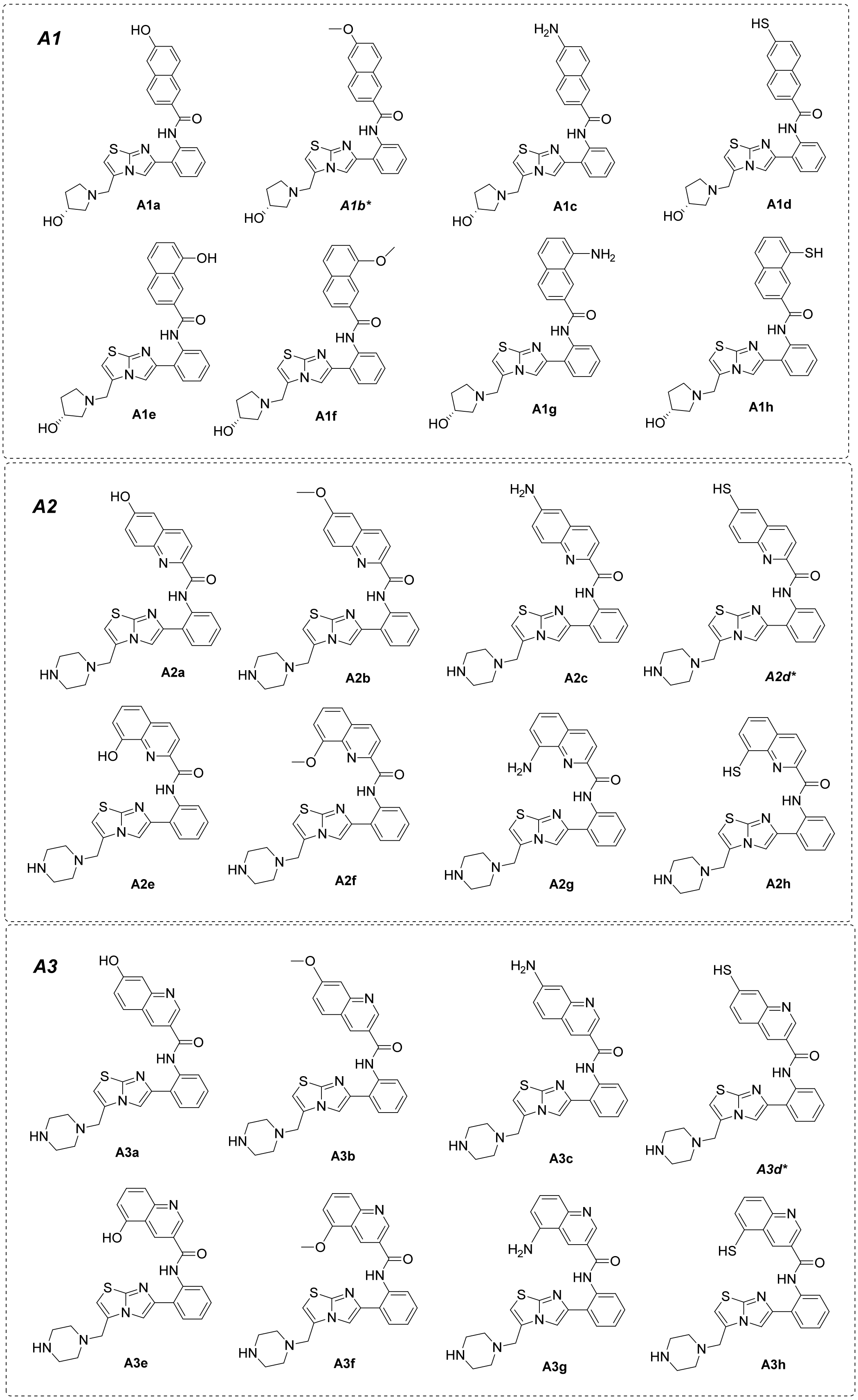

Supplementary Figure 1: Structurally modified compounds **A1-A3** (*The most potent compound in the modified subseries, ** The most potent compound of series A)

EXCLI Journal 2019;18:207-222 – ISSN 1611-2156
Received: March 12, 2019, accepted: March 20, 2019, published: April 05, 2019

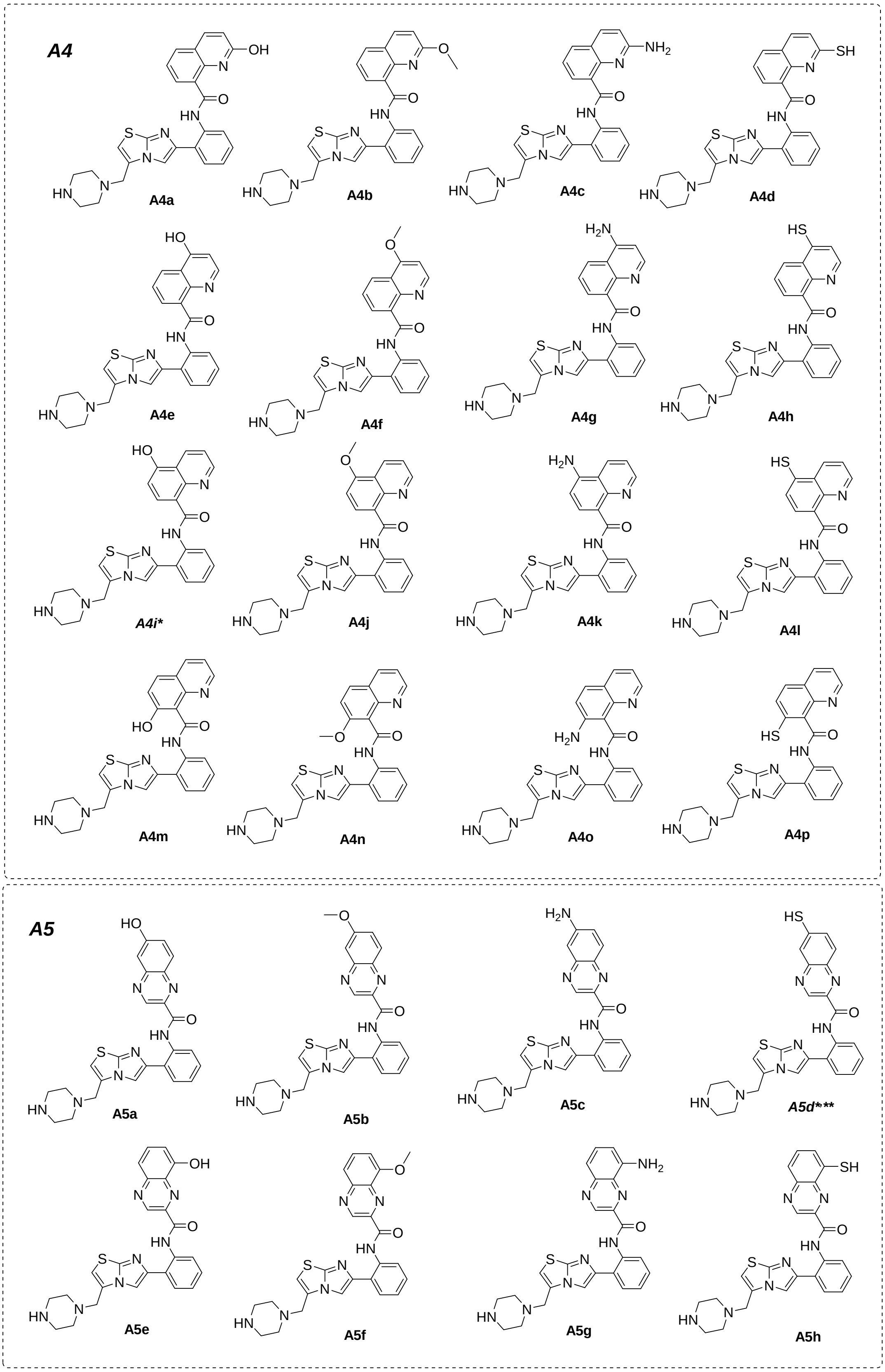

Supplementary Figure S1 (cont.): Structurally modified compounds **A4-A5** (*The most potent compound in the modified subseries, ** The most potent compound of series A)

EXCLI Journal 2019;18:207-222 – ISSN 1611-2156
Received: March 12, 2019, accepted: March 20, 2019, published: April 05, 2019

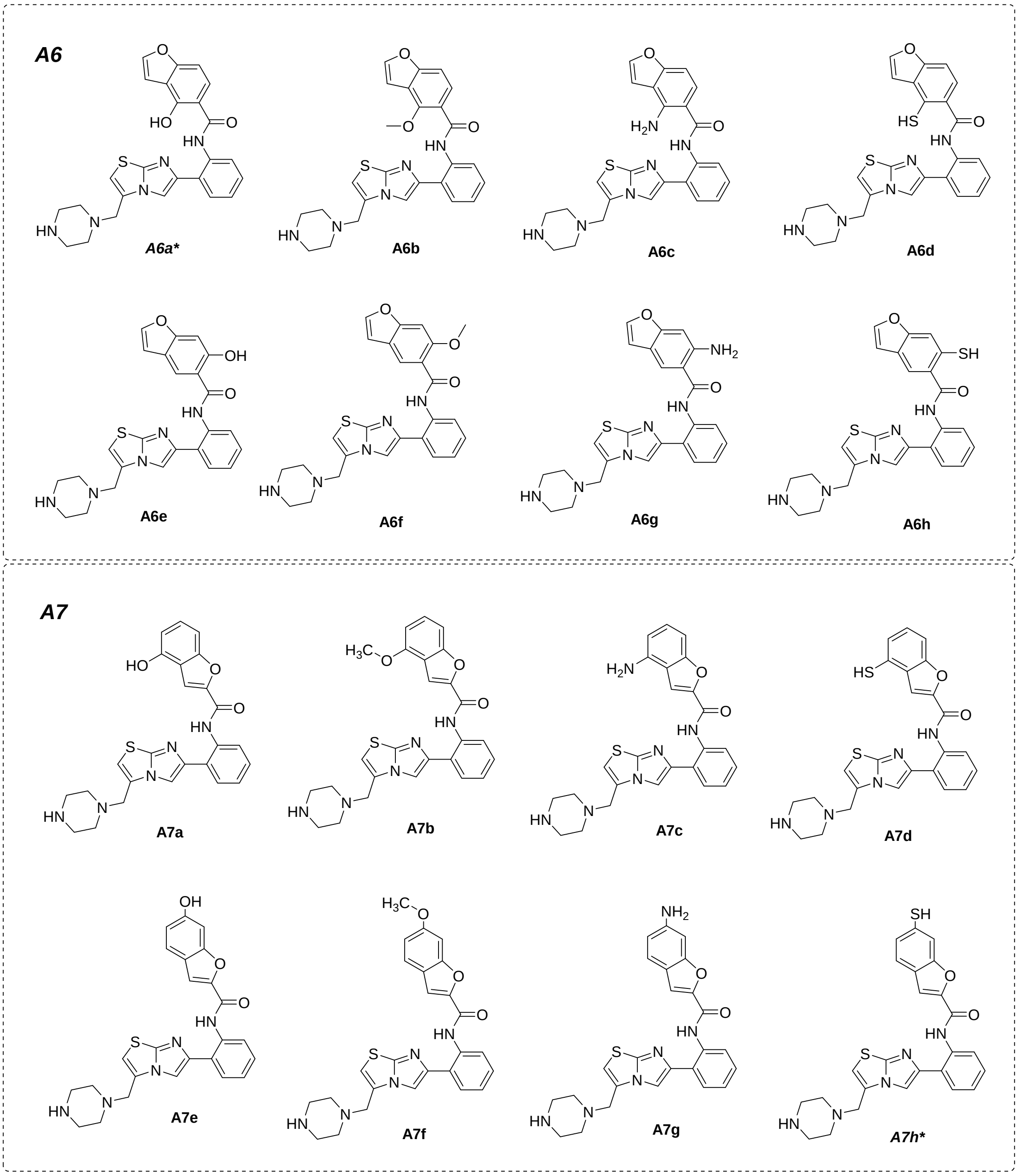

Supplementary Figure 1 (cont.): Structurally modified compounds **A6-A7** (*The most potent compound in the modified subseries, ** The most potent compound of series A)

EXCLI Journal 2019;18:207-222 – ISSN 1611-2156
Received: March 12, 2019, accepted: March 20, 2019, published: April 05, 2019

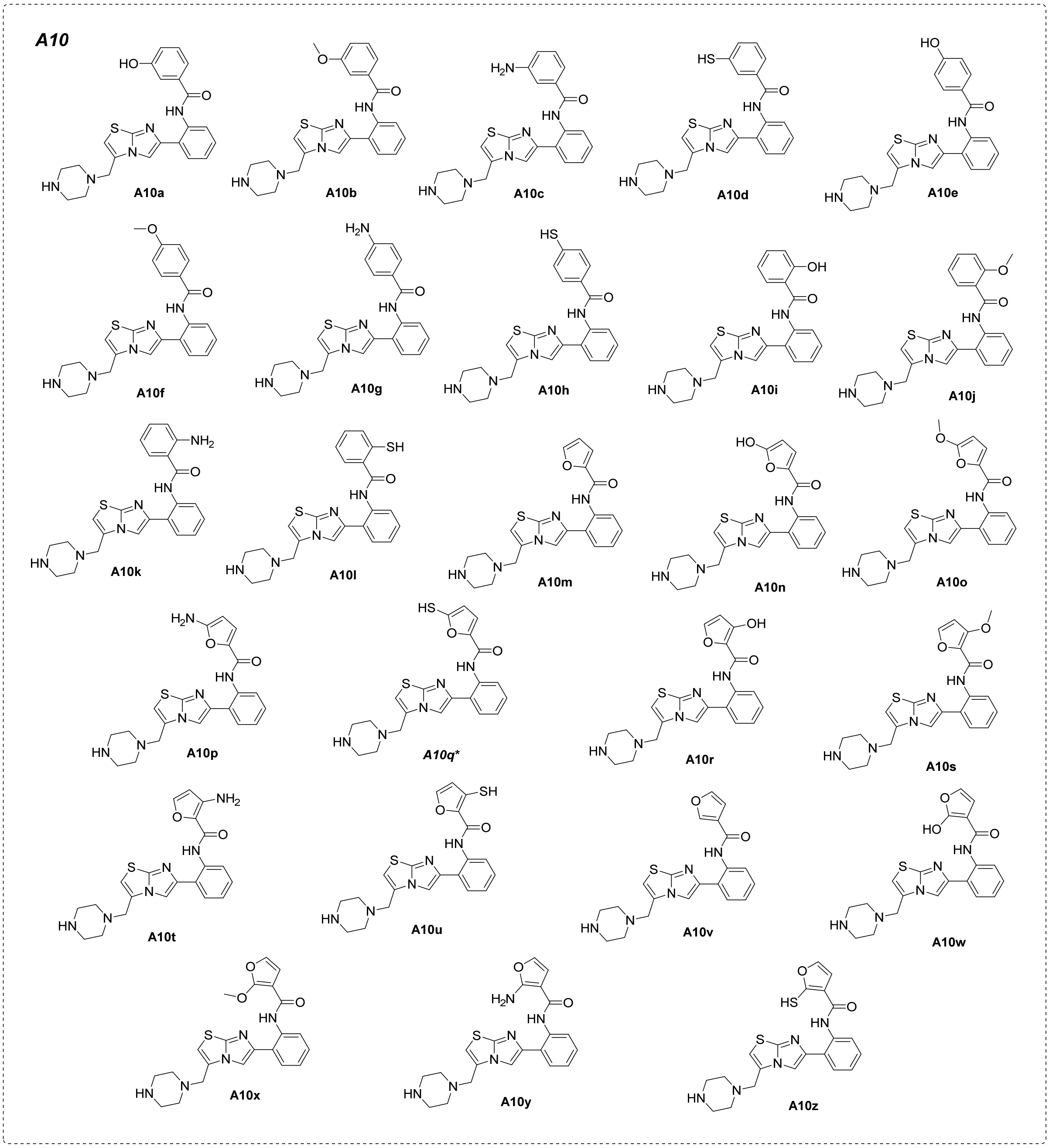

Supplementary Figure 1 (cont.): Structurally modified compounds **A10** (*The most potent compound in the modified subseries, ** The most potent compound of series A)

EXCLI Journal 2019;18:207-222 – ISSN 1611-2156
Received: March 12, 2019, accepted: March 20, 2019, published: April 05, 2019

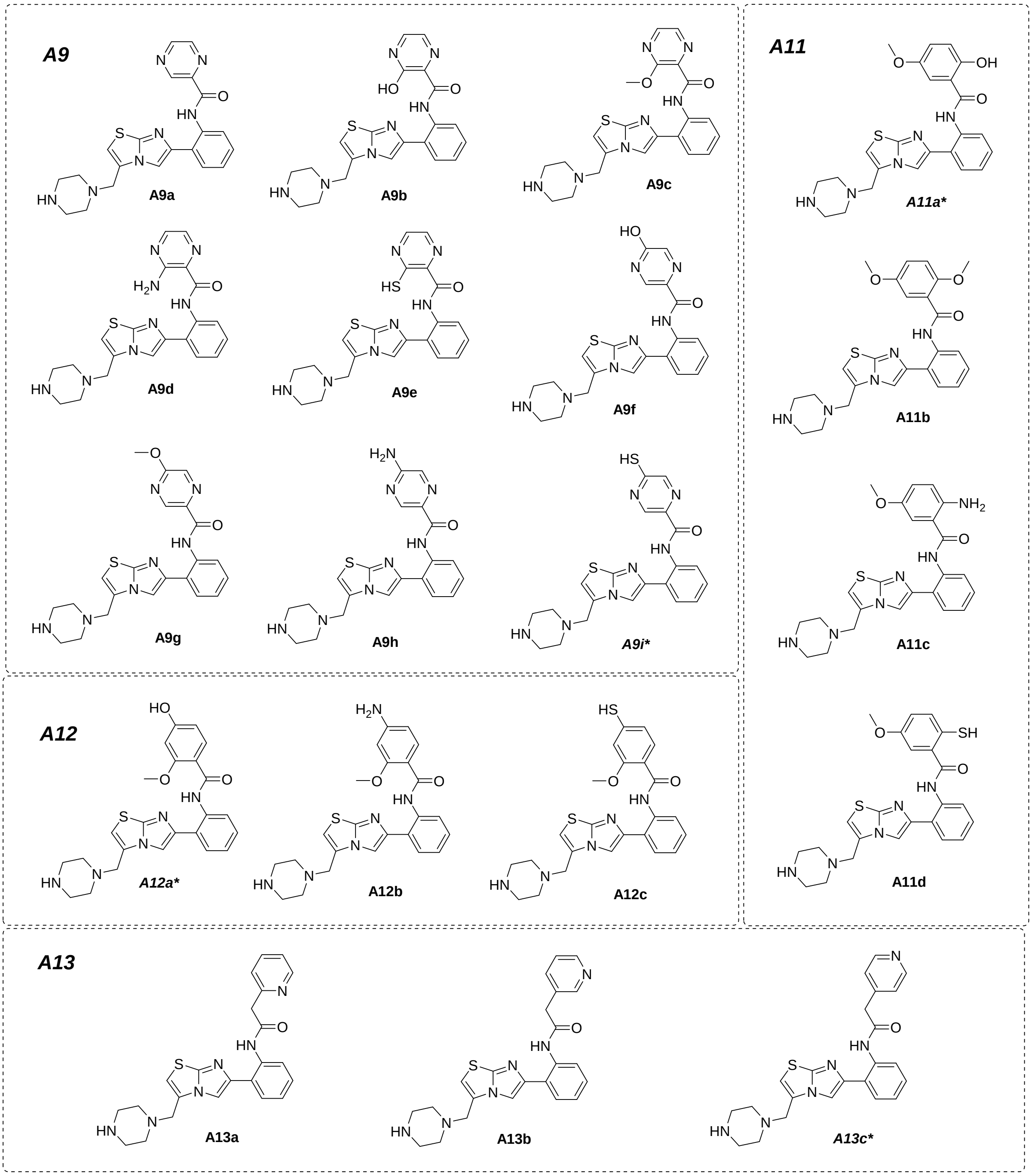

Supplementary Figure 1 (cont.): Structurally modified compounds **A9** and **A11-A13** (*The most potent compound in the modified subseries, ** The most potent compound of series A)

EXCLI Journal 2019;18:207-222 – ISSN 1611-2156
Received: March 12, 2019, accepted: March 20, 2019, published: April 05, 2019

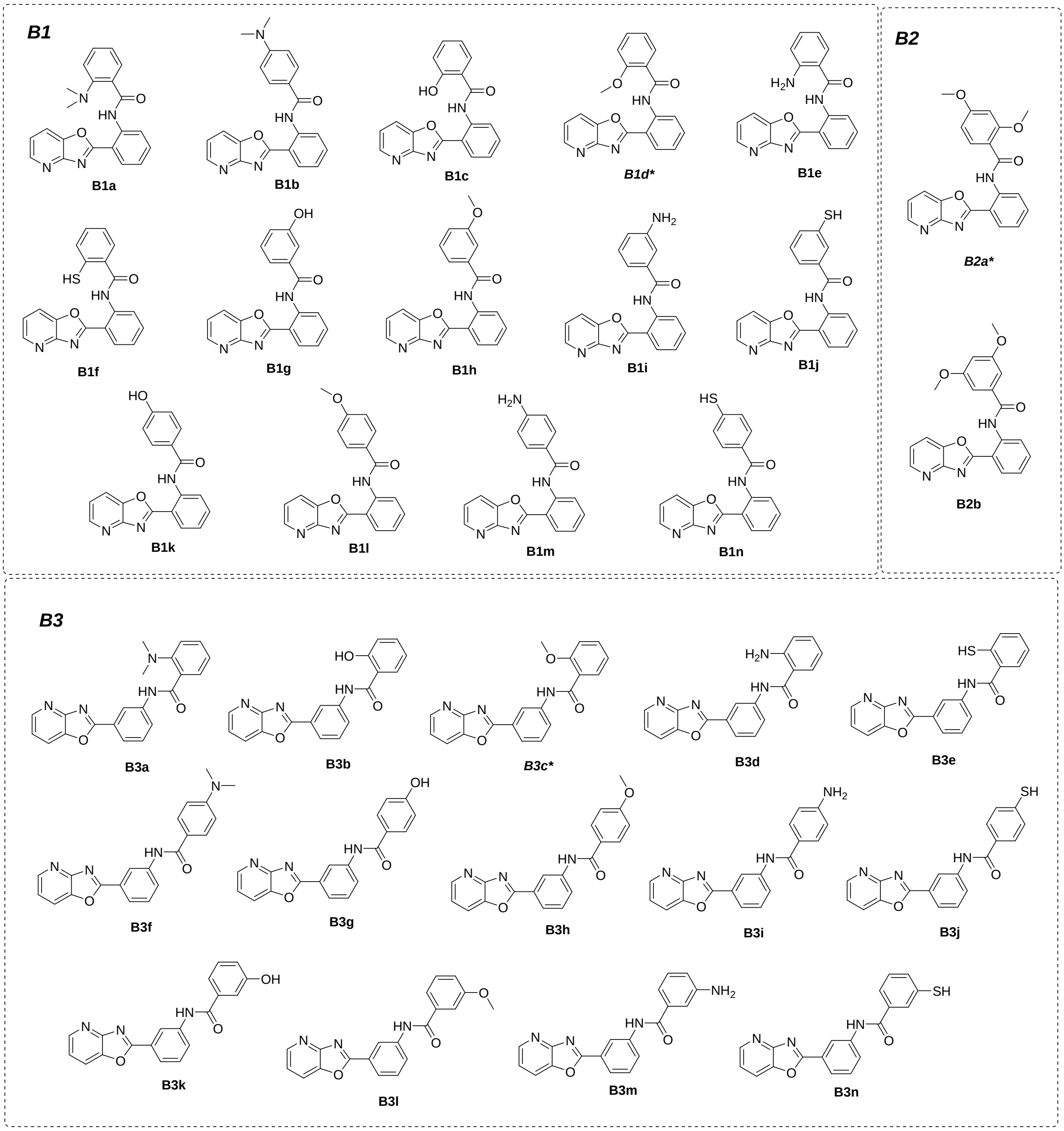

Supplementary Figure 2: Structurally modified compounds **B1-B3** (*The most potent compound in the modified subseries, ** The most potent compound of series B)

EXCLI Journal 2019;18:207-222 – ISSN 1611-2156
Received: March 12, 2019, accepted: March 20, 2019, published: April 05, 2019

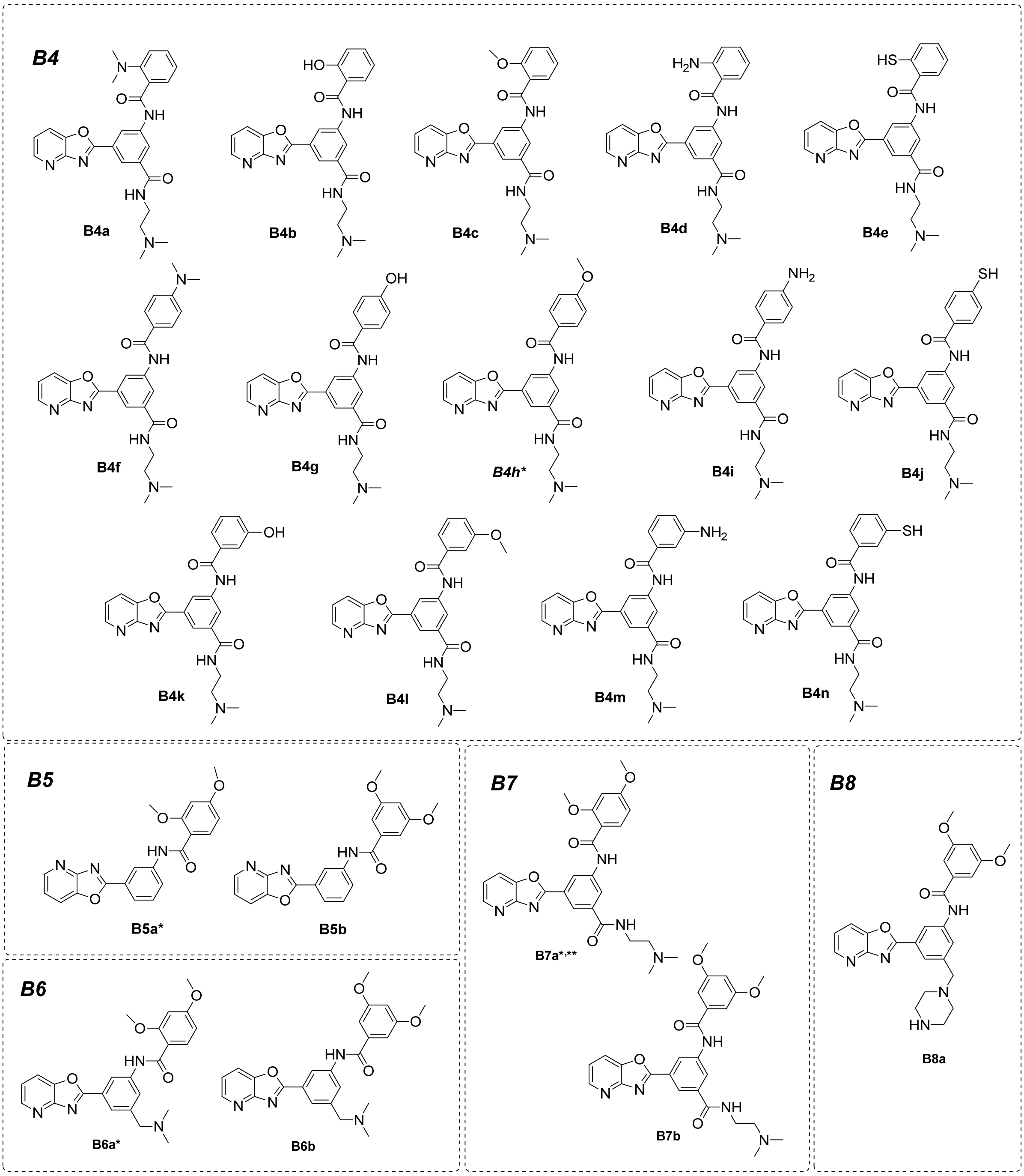

Supplementary Figure 2 (cont.): Structurally modified compounds **B4-B8** (*The most potent compound in the modified subseries, ** The most potent compound of series B)

EXCLI Journal 2019;18:207-222 – ISSN 1611-2156
Received: March 12, 2019, accepted: March 20, 2019, published: April 05, 2019

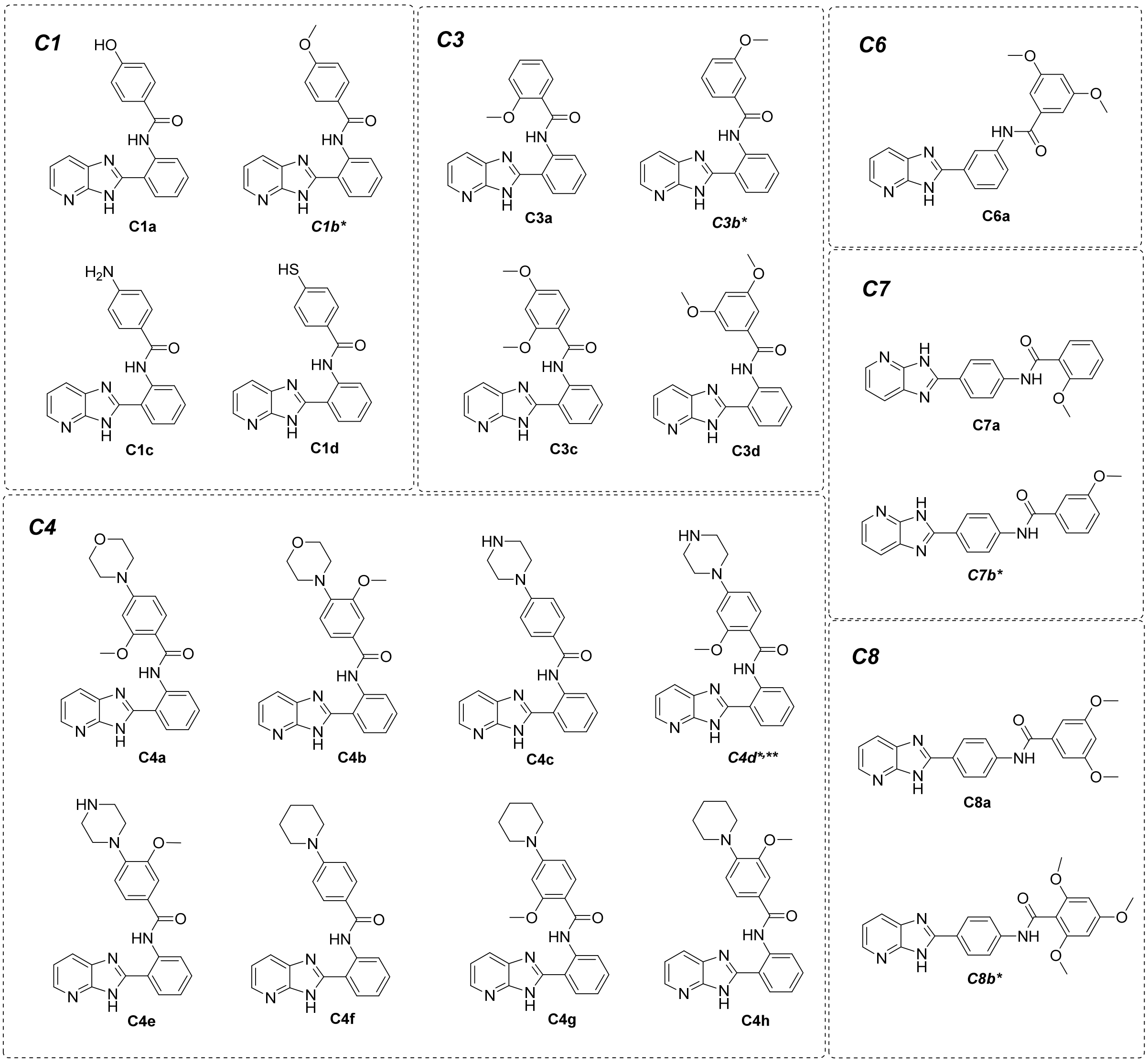

Supplementary Figure 3: Structurally modified compounds **C1-C8** (*The most potent compound in the modified subseries, ** The most potent compound of series C)

EXCLI Journal 2019;18:207-222 – ISSN 1611-2156
Received: March 12, 2019, accepted: March 20, 2019, published: April 05, 2019

Supplementary Table 4: Descriptors values and predicted $pEC_{1.5}$ of modified SIRT1 activators Scaffold A

Compound	HATS8u	Electronegativity	JGI7	Mor15p	Predicted $pEC_{1.5}$
**A1a**	0.181	-0.119	0.012	1.229	-2.170
**A1b**	0.182	-0.118	0.013	1.291	**-1.703***
**A1c**	0.176	-0.113	0.012	1.205	-2.487
**A1d**	0.170	-0.119	0.012	1.231	-2.087
**A1e**	0.183	-0.118	0.011	1.286	-2.891
**A1f**	0.176	-0.117	0.011	1.457	-2.985
**A1g**	0.177	-0.113	0.011	1.204	-3.091
**A1h**	0.169	-0.122	0.011	1.183	-2.447
**A2a**	0.167	-0.124	0.012	1.322	-1.740
**A2b**	0.166	-0.124	0.012	1.342	-1.780
**A2c**	0.171	-0.120	0.012	1.181	-1.994
**A2d**	0.171	-0.129	0.012	1.367	**-1.548***
**A2e**	0.181	-0.123	0.011	1.187	-2.507
**A2f**	0.176	-0.125	0.010	0.971	-2.753
**A2g**	0.170	-0.117	0.011	1.143	-2.694
**A2h**	0.171	-0.129	0.011	1.279	-2.082
**A3a**	0.180	-0.126	0.012	1.122	-1.647
**A3b**	0.167	-0.126	0.012	1.210	-1.595
**A3c**	0.174	-0.121	0.012	1.102	-1.896
**A3d**	0.178	-0.131	0.012	1.207	**-1.387***
**A3e**	0.165	-0.131	0.011	1.256	-1.850
**A3f**	0.220	-0.132	0.010	1.649	-3.195
**A3g**	0.163	-0.125	0.011	1.151	-2.146
**A3h**	0.157	-0.129	0.011	1.345	-1.936
**A4a**	0.322	-0.128	0.010	0.976	-4.176
**A4b**	0.295	-0.121	0.010	0.847	-4.255
**A4c**	0.310	-0.123	0.010	1.199	-4.511
**A4d**	0.245	-0.128	0.010	1.060	-3.391
**A4e**	0.278	-0.127	0.010	1.067	-3.831
**A4f**	0.261	-0.122	0.009	1.285	-4.687
**A4g**	0.274	-0.121	0.010	1.087	-4.138
**A4h**	0.251	-0.132	0.010	1.288	-3.359
**A4i**	0.280	-0.124	0.010	1.153	-4.087
**A4j**	0.251	-0.122	0.010	1.317	-3.984
**A4k**	0.269	-0.122	0.010	1.146	-4.095
**A4l**	0.308	-0.135	0.014	0.824	**-1.107***
**A4m**	0.275	-0.118	0.010	1.334	-4.479
**A4n**	0.323	-0.118	0.010	1.513	-5.154
**A4o**	0.290	-0.119	0.010	1.245	-4.541
**A4p**	0.232	-0.126	0.010	1.073	-3.381

*****The most potent compounds in subseries, ** The most potent compound of series

EXCLI Journal 2019;18:207-222 – ISSN 1611-2156
Received: March 12, 2019, accepted: March 20, 2019, published: April 05, 2019

Supplementary Table 4 (cont.): Descriptors values and predicted $pEC_{1.5}$ of modified SIRT1 activators Scaffold A

Compound	HATS8u	Electronegativity	JGI7	Mor15p	Predicted $pEC_{1.5}$
**A5a**	0.156	-0.134	0.012	1.217	-0.965
**A5b**	0.152	-0.134	0.012	1.334	-1.009
**A5c**	0.150	-0.129	0.012	1.226	-1.231
**A5d**	0.155	-0.138	0.012	1.215	**-0.697*,** **
**A5e**	0.160	-0.132	0.011	1.178	-1.675
**A5f**	0.159	-0.135	0.010	1.259	-2.111
**A5g**	0.156	-0.129	0.011	1.261	-1.879
**A5h**	0.160	-0.117	0.011	1.213	-2.654
**A6a**	0.203	-0.112	0.011	0.912	**-3.249***
**A6b**	0.225	-0.111	0.011	1.017	-3.604
**A6c**	0.241	-0.114	0.011	0.930	-3.533
**A6d**	0.255	-0.116	0.011	1.108	-3.692
**A6e**	0.206	-0.111	0.011	1.006	-3.429
**A6f**	0.279	-0.108	0.011	0.925	-4.338
**A6g**	0.232	-0.113	0.011	0.945	-3.507
**A6h**	0.235	-0.117	0.011	1.004	-3.311
**A7a**	0.176	-0.117	0.011	0.858	-2.635
**A7b**	0.148	-0.114	0.010	0.952	-3.120
**A7c**	0.194	-0.122	0.011	0.833	-2.458
**A7d**	0.170	-0.118	0.011	1.078	-2.622
**A7e**	0.184	-0.116	0.010	0.931	-3.368
**A7f**	0.176	-0.111	0.011	1.096	-3.126
**A7g**	0.182	-0.123	0.010	0.906	-2.910
**A7h**	0.148	-0.129	0.010	1.045	**-2.282***
**A9a**	0.219	-0.130	0.009	0.641	-3.325
**A9b**	0.226	-0.123	0.010	0.818	-3.342
**A9c**	0.253	-0.123	0.010	0.840	-3.670
**A9d**	0.277	-0.127	0.010	0.746	-3.579
**A9e**	0.285	-0.125	0.010	0.627	-3.754
**A9f**	0.219	-0.121	0.010	0.544	-3.236
**A9g**	0.241	-0.117	0.010	0.634	-3.761
**A9h**	0.209	-0.130	0.010	0.795	-2.713
**A9i**	0.193	-0.138	0.010	0.672	**-1.932***
**A10a**	0.243	-0.117	0.010	0.779	-3.891
**A10b**	0.220	-0.116	0.010	0.854	-3.770
**A10c**	0.236	-0.112	0.010	0.789	-4.108
**A10d**	0.244	-0.119	0.010	0.800	-3.747
**A10e**	0.231	-0.113	0.010	0.844	-4.036
**A10f**	0.205	-0.112	0.010	0.883	-3.829
**A10g**	0.195	-0.110	0.010	0.713	-3.746

*The most potent compounds in subseries, ** The most potent compound of series

EXCLI Journal 2019;18:207-222 – ISSN 1611-2156
Received: March 12, 2019, accepted: March 20, 2019, published: April 05, 2019

Supplementary Table 4 (cont.): Descriptors values and predicted $pEC_{1.5}$ of modified SIRT1 activators Scaffold A

Compound	HATS8u	Electronegativity	JGI7	Mor15p	Predicted $pEC_{1.5}$
**A10h**	0.188	-0.118	0.010	1.047	-3.363
**A10i**	0.236	-0.121	0.010	0.731	-3.532
**A10j**	0.269	-0.112	0.010	0.749	-4.479
**A10k**	0.276	-0.113	0.010	0.761	-4.448
**A10l**	0.295	-0.114	0.010	0.843	-4.649
**A10m**	0.355	-0.123	0.009	0.812	-5.309
**A10n**	0.249	-0.111	0.011	0.659	-3.625
**A10o**	0.221	-0.109	0.010	0.736	-4.109
**A10p**	0.240	-0.105	0.011	0.610	-3.893
**A10q**	0.249	-0.119	0.011	0.501	**-3.054***
**A10r**	0.267	-0.117	0.011	0.759	-3.506
**A10s**	0.248	-0.108	0.010	0.755	-4.454
**A10t**	0.277	-0.110	0.011	0.715	-4.044
**A10u**	0.293	-0.117	0.011	0.825	-3.835
**A10v**	0.228	-0.115	0.009	0.697	-4.413
**A10w**	0.284	-0.116	0.011	0.775	-3.817
**A10x**	0.276	-0.108	0.010	0.649	-4.728
**A10y**	0.293	-0.107	0.011	0.622	-4.325
**A10z**	0.300	-0.116	0.011	0.654	-3.877
**A11a**	0.195	-0.114	0.011	1.404	**-3.353***
**A11b**	0.266	-0.112	0.010	1.144	-4.694
**A11c**	0.263	-0.107	0.011	1.041	-4.290
**A11d**	0.247	-0.114	0.011	1.341	-3.890
**A12a**	0.236	-0.114	0.011	1.094	-3.582
**A12b**	0.222	-0.109	0.011	1.051	-3.702
**A12c**	0.234	-0.116	0.011	1.167	**-3.473***
**A13a**	0.259	-0.113	0.010	0.652	-4.200
**A13b**	0.220	-0.118	0.010	0.663	-3.491
**A13c**	0.223	-0.119	0.010	0.496	**-3.361***

*****The most potent compounds in subseries, ** The most potent compound of series

Received: March 12, 2019, accepted: March 20, 2019, published: April 05, 2019

Supplementary Table 5: Descriptors values and predicted $pEC_{1.5}$ of modified SIRT1 activators Scaffold B

Compound	Mor22e	F10[C-O]	P1e	Predicted $pEC_{1.5}$
**B1a**	-0.043	0	0.700	-3.812
**B1b**	0.042	0	0.672	-3.831
**B1c**	-0.039	1	0.611	-3.533
**B1d**	-0.407	1	0.566	**-3.101***
**B1e**	-0.104	0	0.620	-3.588
**B1f**	0.130	0	0.600	-3.761
**B1g**	0.086	2	0.620	-3.571
**B1h**	-0.138	3	0.689	-3.415
**B1i**	0.089	0	0.558	-3.635
**B1j**	0.226	0	0.537	-3.716
**B1k**	0.165	1	0.574	-3.642
**B1l**	-0.138	1	0.683	-3.594
**B1m**	-0.027	0	0.605	-3.627
**B1n**	-0.052	0	0.594	-3.581
**B2a**	-0.640	2	0.591	**-2.845***
**B2b**	0.183	6	0.603	-3.242
**B3a**	0.193	5	0.807	-3.776
**B3b**	0.183	5	0.864	-3.886
**B3c**	0.055	6	0.822	**-3.585***
**B3d**	0.007	3	0.867	-3.923
**B3e**	0.139	3	0.821	-3.947
**B3f**	0.073	3	0.856	-3.960
**B3g**	0.205	5	0.852	-3.881
**B3h**	-0.064	5	0.875	-3.683
**B3i**	0.084	3	0.867	-3.993
**B3j**	0.184	3	0.861	-4.072
**B3k**	0.279	3	0.849	-4.134
**B3l**	-0.081	3	0.878	-3.865
**B3m**	0.188	3	0.872	-4.099
**B3n**	0.079	3	0.871	-3.997

***The most potent compounds in subseries, ** The most potent compound of series

EXCLI Journal 2019;18:207-222 – ISSN 1611-2156
Received: March 12, 2019, accepted: March 20, 2019, published: April 05, 2019

Supplementary Table 5 (cont.): Descriptors values and predicted $pEC_{1.5}$ of modified SIRT1 activators Scaffold B

Compound	Mor22e	F10[C-O]	P1e	Predicted $pEC_{1.5}$
**B4a**	-0.043	10	0.543	-2.526
**B4b**	-0.439	9	0.555	-2.284
**B4c**	-0.187	11	0.677	-2.581
**B4d**	-0.121	6	0.648	-3.058
**B4e**	-0.084	6	0.601	-2.993
**B4f**	-0.477	6	0.651	-2.738
**B4g**	-0.154	9	0.545	-2.524
**B4h**	-0.468	9	0.552	**-2.251***
**B4i**	-0.174	6	0.568	-2.841
**B4j**	-0.049	6	0.524	-2.863
**B4k**	-0.107	6	0.587	-2.943
**B4l**	-0.239	6	0.568	-2.782
**B4m**	0.172	6	0.708	-3.453
**B4n**	-0.268	6	0.591	-2.803
**B5a**	-0.579	8	0.863	**-2.899***
**B5b**	-0.162	3	0.831	-3.692
**B6a**	-0.624	11	0.698	**-2.224***
**B6b**	-0.579	3	0.709	-3.053
**B7a**	-0.786	14	0.603	**-1.589*,****
**B7b**	-0.869	6	0.523	-2.110
**B8a**	-0.275	11	0.572	-2.279

*The most potent compounds in subseries, ** The most potent compound of series

EXCLI Journal 2019;18:207-222 – ISSN 1611-2156
Received: March 12, 2019, accepted: March 20, 2019, published: April 05, 2019

Supplementary Table 6: Descriptors values and predicted $pEC_{1.5}$ of modified SIRT1 activators Scaffold C

Compound	RDF090u	BEHp2	R6m+	E2u	Predicted $pEC_{1.5}$
**C1a**	12.513	3.805	0.019	0.433	-2.410
**C1b**	13.246	3.808	0.032	0.424	**-2.167***
**C1c**	14.867	3.812	0.016	0.435	-2.465
**C1d**	11.672	3.830	0.038	0.431	-2.584
**C3a**	8.376	3.809	0.015	0.489	-3.085
**C3b**	17.390	3.809	0.016	0.440	**-2.305***
**C3c**	15.710	3.817	0.015	0.509	-2.944
**C3d**	17.005	3.816	0.019	0.501	-2.748
**C4a**	23.539	3.837	0.016	0.373	-2.086
**C4b**	20.759	3.837	0.015	0.378	-2.280
**C4c**	20.679	3.831	0.014	0.371	-2.154
**C4d**	28.043	3.838	0.013	0.380	**-1.948*,****
**C4e**	19.962	3.838	0.016	0.385	-2.364
**C4f**	18.278	3.834	0.014	0.347	-2.200
**C4g**	19.710	3.841	0.016	0.452	-2.812
**C4h**	29.155	3.841	0.014	0.454	-2.345
**C6a**	10.517	3.818	0.018	0.448	-2.848
**C7a**	12.476	3.813	0.012	0.500	-2.687
**C7b**	8.909	3.812	0.017	0.474	**-2.470***
**C8a**	8.474	3.819	0.024	0.414	-3.044
**C8b**	10.973	3.828	0.013	0.341	**-2.992***

*The most potent compounds in subseries, ** The most potent compound of series
